# TIM3 activates the ERK1/2 pathway to promote invasion and migration of thyroid tumors

**DOI:** 10.1371/journal.pone.0297695

**Published:** 2024-04-03

**Authors:** Xiao Jin, Zhibo Yin, Xiaoyu Li, Hao Guo, Bo Wang, Shanshan Zhang, Yong Li

**Affiliations:** 1 Department of Thyroid and Breast Surgery, The Second Hospital of Hebei Medical University, Shijiazhuang, China; 2 Department of Ophthalmology, The Second Hospital of Hebei Medical University, Shijiazhuang, China; 3 The Third Department of External Surgery, The Fourth Hospital of Hebei Medical University, Shijiazhuang, China; Kimura Hospital, JAPAN

## Abstract

**Background:**

This study aims to study the possible action mechanism of T-cell immunoglobulin and mucin domain 3 (TIM3) on the migratory and invasive abilities of thyroid carcinoma (TC) cells.

**Methods:**

GSE104005 and GSE138198 datasets were downloaded from the GEO database for identifying differentially expressed genes (DEGs). Functional enrichment analysis and protein-protein interaction (PPI) analysis were performed on the common DEGs in GSE104005 and GSE138198 datasets. Subsequently, in order to understand the effect of a common DEG (TIM3) on TC cells, we performed in vitro experiments using FRO cells. The migratory and invasive abilities of FRO cells were detected by wound scratch assay and Transwell assay. Proteins expression levels of the phosphorylated (p)-extracellular signal-regulated kinase (ERK)1/2, matrix metalloproteinase-2 (MMP-2) and MMP-9 were determined *via* Western blotting after ERK1/2 inhibition in TIM3-NC group and TIM3-mimic group.

**Results:**

316 common DEGs were identified in GSE104005 and GSE138198 datasets. These DEGs were involved in the biological process of ERK1 and ERK2 cascade. TIM3 was significantly up-regulated in TC. In vitro cell experiments showed that TIM3 could promote migration and invasion of TC cells. Moreover, TIM3 may affect the migration, invasive abilities of TC cells by activating the ERK1/2 pathway.

**Conclusion:**

The above results indicate that TIM3 may affect the migratory and invasive of TC cells by activating the ERK1/2 pathway.

## Introduction

Thyroid carcinoma (TC) is a common endocrine cancer, which derived from two types of thyroid endocrine cells, follicular thyroid cells and parafollicular C cells. Papillary thyroid carcinoma, follicular thyroid carcinoma, poorly differentiated thyroid carcinoma and anaplastic thyroid carcinoma derived from follicular thyroid cells account for the majority of thyroid carcinoma. Medullary thyroid carcinoma derived from parafollicular C cells accounts for a small proportion of thyroid carcinoma [1]. The incidence of TC is correlated with genetic abnormalities and environmental factors [2]. The incidence of TC is increasing in different populations worldwide [3]. Surgical resection is the standard treatment for most patients with TC, supplemented by thyrotropin suppression and radioiodine therapy according to the patient’s condition [4]. In recent years, the discovery of novel biomarkers of TC has significantly improved the understanding of the molecular pathogenesis of TC, leading to more personalized treatment for patients with TC. In addition, molecular analysis of cancer is also frequently used for diagnostic purposes, including preoperative fine-needle biopsy specimens, and to identify altered targeted pathways in the disease to guide clinical trials of drug therapy for advanced TC [5]. Therefore, continuous search for potential targets is essential for better treatment of TC.

As an important negative regulator of immune cells, T-cell immunoglobulin and mucin domain 3 (TIM3) was first discovered on differentiated T helper cell type 1 in 2002 [6] and belongs to the TIM family, which is composed of an N-terminal immunoglobulin-like domain [7]. High expression of TIM3 on tumor-infiltrating lymphocytes is associated with poor prognosis of tumors [8, 9]. Besides, TIM3 serves as an independent factor to predict the prognosis of cervical cancer, and down-regulating TIM3 inhibits the migration and invasion of cervical cancer cells [10]. A study suggests the independent prognostic value of TIM3 in medullary thyroid carcinoma and finds that this negative prognostic effect increases with expression levels [11]. In addition, another study showed that TIM3 expression had no prognostic significance in patients with medullary thyroid carcinoma, but was associated with PD-1 expression and perineural invasion in patients with medullary thyroid carcinoma [12]. It is reported that TIM3 induces tumor-promoting M2-like macrophage polarization in anaplastic thyroid carcinoma. Moreover, TIM3 interference could be a potential tool to treat patients with anaplastic thyroid carcinoma [13]. Moreover, TIM3 is increased in patients with differentiated thyroid carcinoma and is associated with cervical lymph node metastasis [14]. In this study, combined with bioinformatics analysis, the influences of TIM3 expression on the migratory, invasive and proliferative abilities of TC cells were detected to analyze its possible mechanism of action, thus laying a foundation for research on potential biological targets of thyroid tumors.

## Materials and methods

### Bioinformatics analysis

Two TC-related datasets involving RNA-seq data, GSE104005 (involving 5 normal controls and 29 cases including papillary thyroid carcinoma, poorly differentiated and anaplastic thyroid carcinoma) and GSE138198 (involving 3 normal controls and 20 cases including papillary carcinoma of thyroid) were downloaded from the Gene Expression Omnibus (GEO) database. The "FactoMineR" and "FactoExtra"R packages in R language were used to conduct the principal component analysis (PCA) for the two groups of samples in each dataset. Then the Limma software package in R language was utilized for quantile normalization. Differentially expressed genes (DEGs) were identified under the threshold value of (|log fold change (FC)| > 1 and *p* < 0.05. Volcano plots and heat maps of DEGs were visualized using the ggplot2 package in R software and the Pheatmap package in R software, respectively.

### Functional enrichment analysis of common DEGs in two datasets

The common DEGs in the datasets GSE104005 and GSE138198 were obtained *via* the RobustRankAggreg algorithm and then subjected to Genome Ontology (GO) and Kyoto Encyclopedia of Genes and Genomes (KEGG) enrichment analysis. Next, the corresponding DEGs were analyzed from the aspects of biological process (BP), cell component (CC) and molecular function (MF) using an online database tool of Database for Annotation, Visualization and Integration Discovery (DAVID). Finally, the GOplot and ggplot2 packages in R language were employed to plot the GO and KEGG enrichment analysis diagram. In addition, gene set enrichment analysis (GSEA) tool was performed in two datasets.

### Protein-protein interaction (PPI) analysis of common DEGs in two datasets

The PPI network analysis was conducted for the common DEGs in two datasets. A PPI network was constructed based on the STRING database. The hub genes were determined by the Cytoscape plugin CytoHubba.

### Cell culture and grouping

To investigate the deeper function of one of the common DEGs (TIM3) at the cellular level, the cell experiment was performed. TC cells (FRO cells) were routinely cultured and passaged in a 37°C incubator with Dulbecco’s modified Eagle medium (DMEM) containing 10% of fetal bovine serum (FBS) under 5% CO_2_ and saturated humidity. The cells in logarithmic growth phase were used for experiment. Specifically, FRO cells were seeded into a 6-well plate at 3×10^5^ cells/well and incubated in the 37°C incubator overnight until they reached 70–80% confluence. The TIM3 overexpression vector was constructed using pcDNA3.1 plasmid. The TIM3 overexpression group was named TIM3-mimic group. si-RNA was used to silence the TIM3 gene, and the TIM3 gene silencing group was named TIM3-inhibitor group. Subsequently, the constructed vector was transfected into FRO cells according to the Lipofectamine 2000 kit (Invitrogen, USA) instructions. Finally, cells were collected 48 h after transfection for follow-up studies. The sequences of primers related to gene overexpression and silencing are shown in the S1 Table, and the overexpression vectors used are shown in the S1 Fig. The present study was approved by the Ethics Committee of The Second Hospital of Hebei Medical University (2021-R022). All methods were carried out in accordance with relevant guidelines and regulations. This study complied with the Declaration of Helsinki. Informed consent was not obtained due to the analysis data came from public datasets.

### Detection of TC cell migration and invasion by Transwell assay

In migration assay, a total of 100 μL of re-suspended serum-free cells were inoculated in the upper Transwell chamber, cultured for 24 h, stained with 0.1% of crystal violet and counted in 5 fields of vision randomly selected under a microscope. In terms of invasion assay, matrigel was plated in the upper Transwell chamber, and the remaining procedures were identical to those in the migration assay.

### Wound scratch assay

Migratory ability of cells was determined through wound-healing assay. Specifically, 1×10^6^ cells were seeded into each well of the 6-well plate and cultured to 90% confluence. Then a wound was made in the central region of the cell monolayer by scratching the plate *via* the tip of a 10 μL pipette. After discarding of culture solution and rinsing with phosphate-buffered saline (PBS) twice, each well was added with serum-free DMEM. Images of migration area were captured using an optical microscope at 0, 12 and 24 h.

### Western blotting

The collected protein samples were lysed by RIPA lysis buffer and centrifuged to acquire the supernatant. The standard curve of proteins was plotted using the BCA kit to measure the concentration of protein samples. Next, the additional amount of the system composed of 5× loading buffer and PBS was calculated, and the proteins were denatured at 100°C for 10 min. After that, the proteins were separated by 10% of SDS-PAGE gel and transferred onto a PVDF membrane, followed by sealing with 5% of BSA for 2 h. The membrane was incubated with relevant primary antibodies on a shaking table at 4°C overnight. The next day, corresponding secondary antibodies were added for 2 h of incubation. The proteins were exposed by color developer in an exposure machine, and images were collected finally.

### Statistical analysis

SPSS 20.00 was used for statistical analysis. Measurement data as mainly observed data were expressed by mean ± SD. The *t*-test was utilized for comparison between two groups, and one-way analysis of variance for comparison among multiple groups. The *p* < 0.05 suggested that the difference was statistically significant. The prism is used to graph.

## Results

### Screening of DEGs in TC

**Fig 1:**
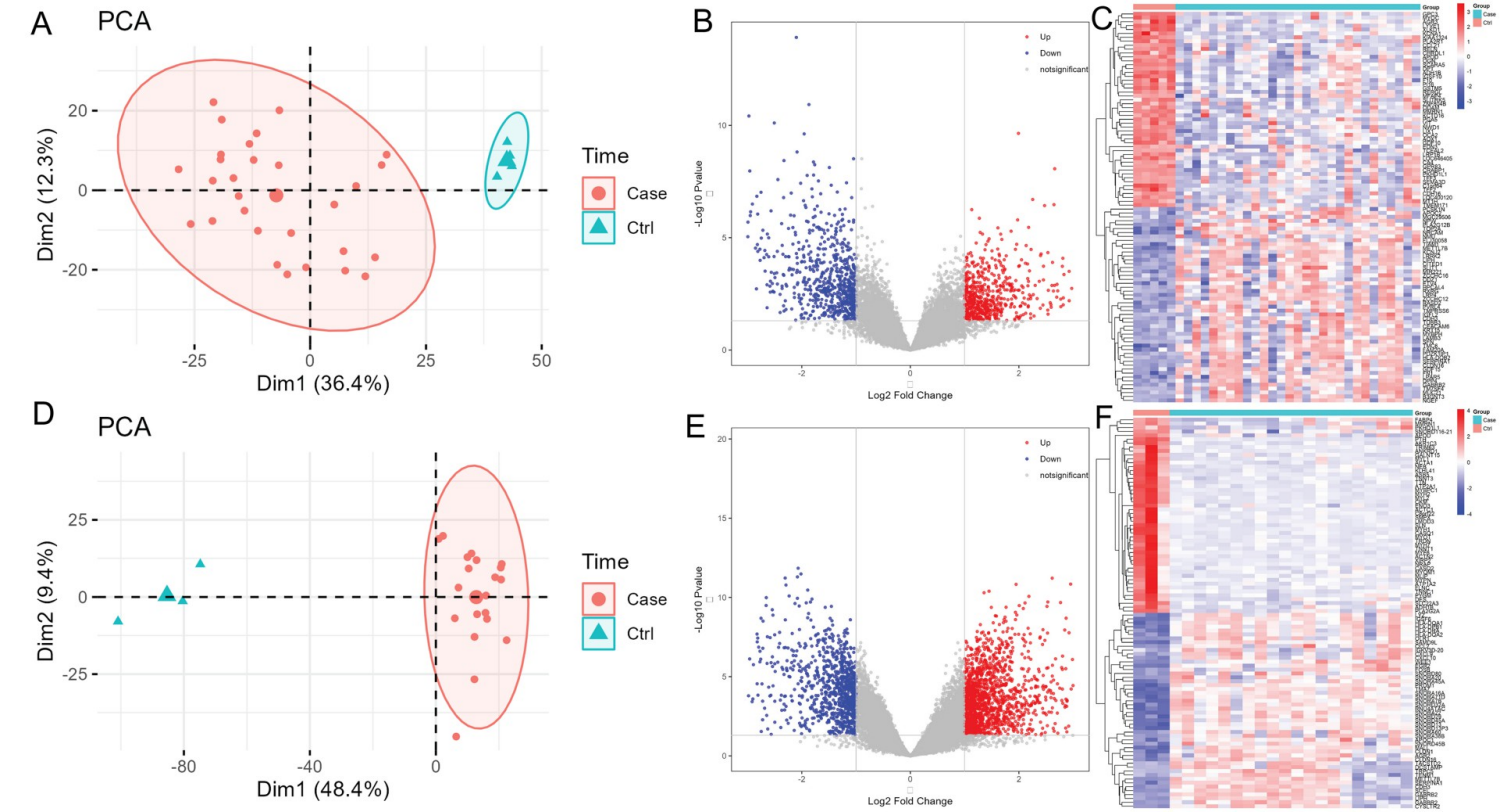
The PCA diagrams, volcano plots and heat maps of DEGs in the GSE104005 and GSE138198 datasets. A: PCA diagram of DEGs in the GSE104005 dataset. Red circles and cyan triangles represent case and control samples, respectively. B: Volcano plot of DEGs in the GSE104005 dataset. X-axis and Y-axis presents -log10 (P-value) and log2Fold Change, respectively. Blue and red color represents down-regulated and up-regulated, respectively. C: Heat map of DEGs in the GSE104005 dataset. Red indicates above the reference channel. Blue indicates below the reference channel. D: PCA diagram of DEGs in the GSE138198 dataset. Red circles and cyan triangles represent case and control samples, respectively. E: Volcano plot of DEGs in the GSE138198 dataset. X-axis and Y-axis presents -log10 (P-value) and log2Fold Change, respectively. Blue and red color represents down-regulated and up-regulated, respectively. F: Heat map of DEGs in the GSE138198 dataset. Red indicates above the reference channel. Blue indicates below the reference channel.

A total of 1252 DEGs (573 up-regulated and 679 down-regulated genes) and 2359 DEGs (1400 up-regulated and 959 down-regulated genes) were obtained from the GSE104005 and GSE138198 datasets, respectively. PCA results showed that the two groups in each dataset were clustered into different clusters. The PCA diagrams, volcano plots and heat maps of DEGs in the GSE104005 and GSE138198 datasets are shown in Fig 1A–1F, respectively. Totally, 316 common DEGs were identified in GSE104005 and GSE138198 datasets. Among which, TIM3, significantly up-regulated in TC compared with normal controls, was regarded as the focus of this study.

### Functional enrichment analysis of 316 common DEGs

**Fig 2:**
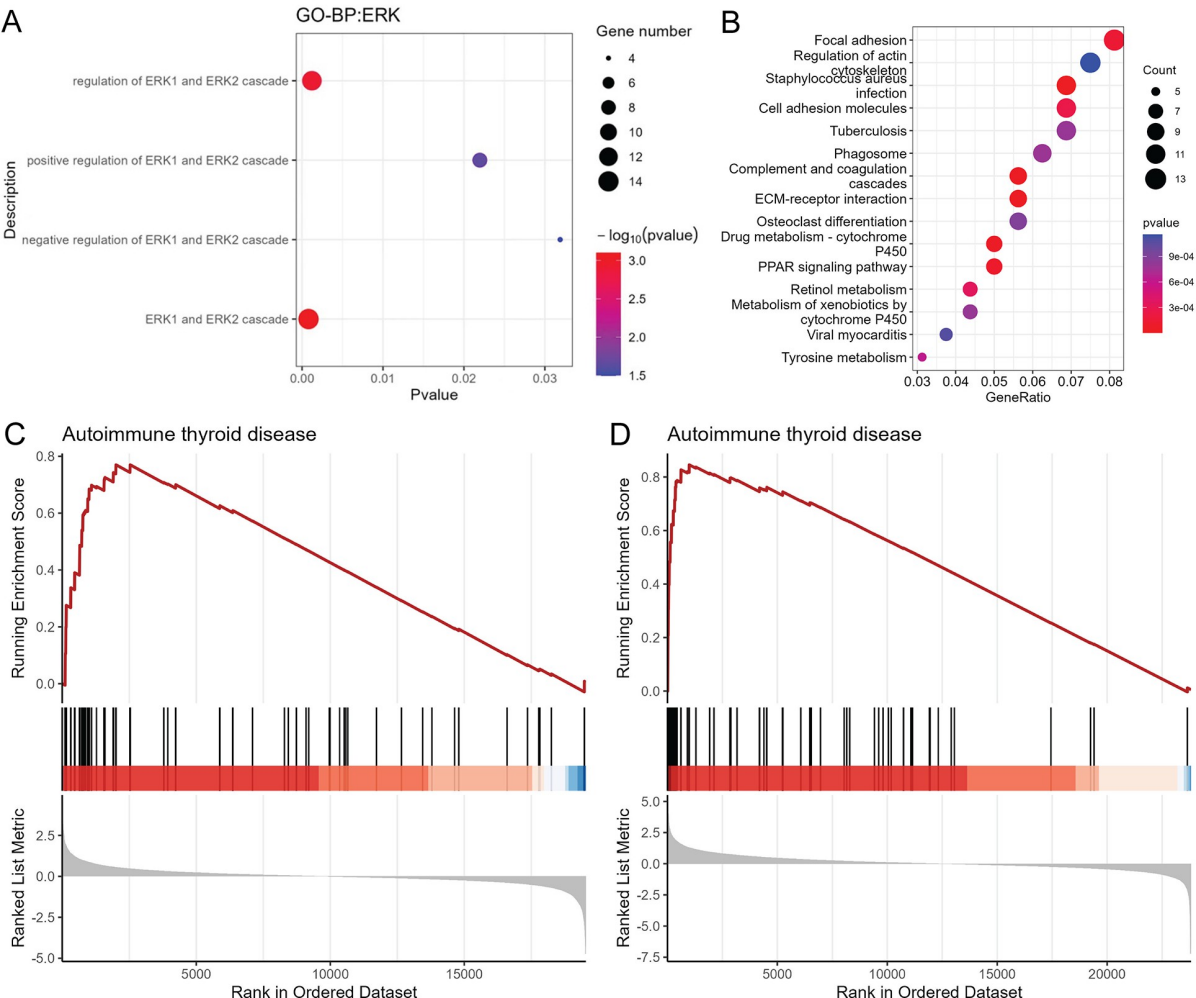
Functional enrichment analysis of 316 common DEGs in TC. A: ERK related pathways enriched in the BP terms of GO analysis; B: KEGG functional enrichment of common DEGs; C: GSEA showed that autoimmune thyroid disease was enriched in the GSE104005 dataset; D: GSEA showed that autoimmune thyroid disease was enriched in the GSE138198 dataset.

Based on BP terms of GO analysis, 316 common DEGs were involved in ERK1 and ERK2 cascade, regulation of ERK1 and ERK2 cascade, positive regulation of ERK1 and ERK2 cascade, and negative regulation of ERK1 and ERK2 cascade (Fig 2A). In the KEGG analysis, staphylococcus aureus infection, complement and coagulation cascades and ECM-receptor interaction were the most significantly enriched signaling pathways (Fig 2B). Additionally, the GSEA results showed that autoimmune thyroid disease was enriched in GSE104005 dataset (Fig 2C) and GSE138198 dataset (Fig 2D). It is reported that autoimmune thyroid disease is associated with the development of TC [15].

### PPI network analysis of 316 common DEGs

**Fig 3:**
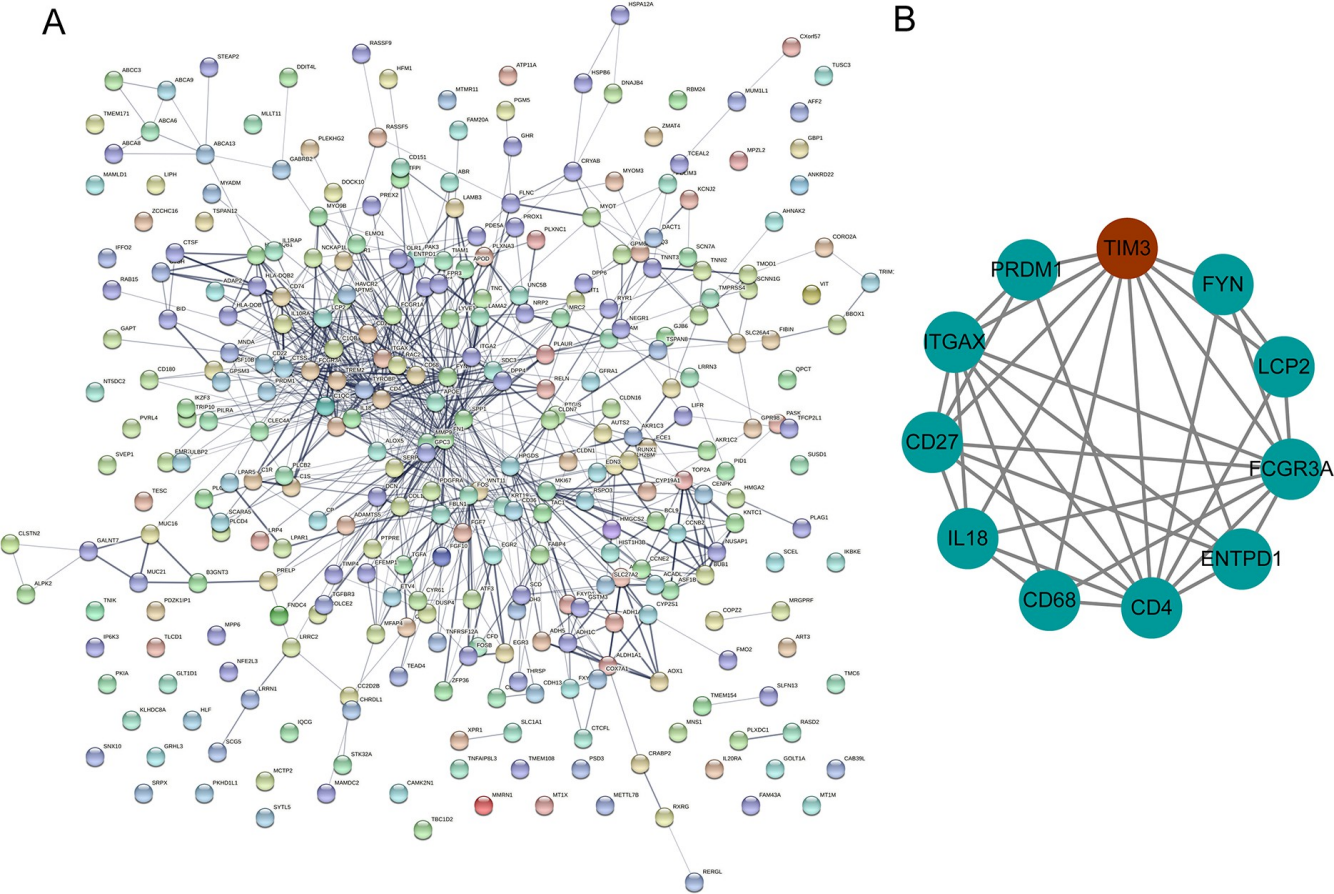
PPI network analysis of 316 common DEGs in TC. A: Total PPI network of 316 common DEGs was visualized by Cytoscape software; B: PPI sub-network containing TIM3 was visualized by Cytoscape software.

The PPI network analysis was conducted for the 316 common DEGs (Fig 3A). It is noted that 10 DEGs had an interaction with TIM3 (Fig 3B), including PR/SET domain 1 (PRDM1), integrin subunit alpha X (ITGAX), CD27 molecule (CD27), interleukin 18 (IL18), CD68 molecule (CD68), CD4 molecule (CD4), ectonucleoside triphosphate diphosphohydrolase 1 (ENTPD1), Fc gamma receptor IIIa (FCGR3A), lymphocyte cytosolic protein 2 (LCP2), and FYN proto-oncogene, Src family tyrosine kinase (FYN).

### TIM3 promoted migration and invasion of TC cells

**Fig 4:**
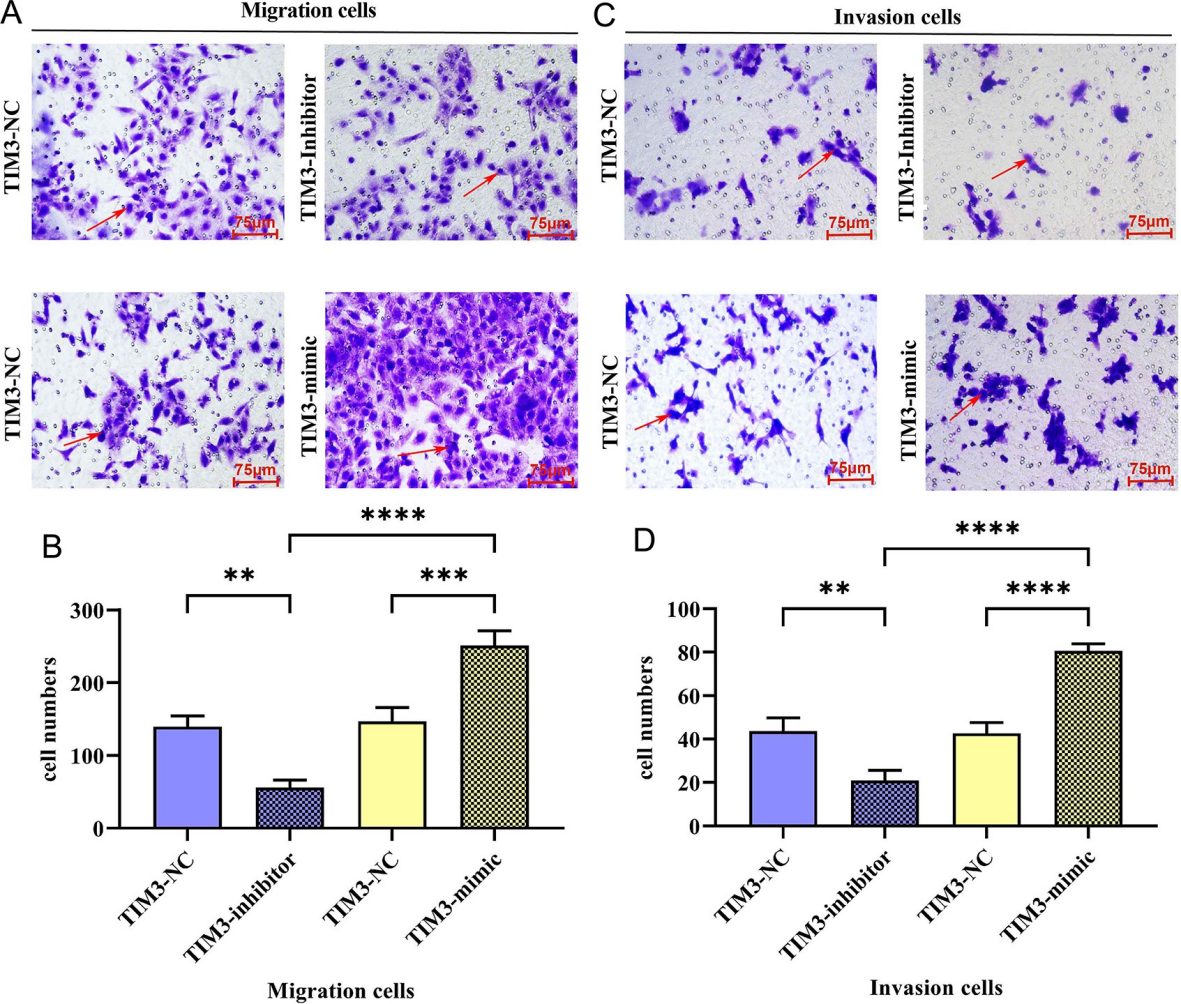
Detection of the number of migrating and invasive TC cells by Transwell migration and invasion experiments. A: Transwell migration experiment was used to detect the migration of TC cell in TIM3-inhibitor and TIM3-mimic groups (×400); B: Statistical analysis of the migration of TC cell in TIM3-inhibitor and TIM3-mimic groups; C: Transwell invasion experiment was used to detect the invasion of TC cell in TIM3-inhibitor and TIM3-mimic groups (×400); D: Statistical analysis of the invasion of TC cell in TIM3-inhibitor and TIM3-mimic groups. ** represents *p* < 0.01, *** represents *p* < 0.001, **** represents *p* < 0.0001.

**Fig 5:**
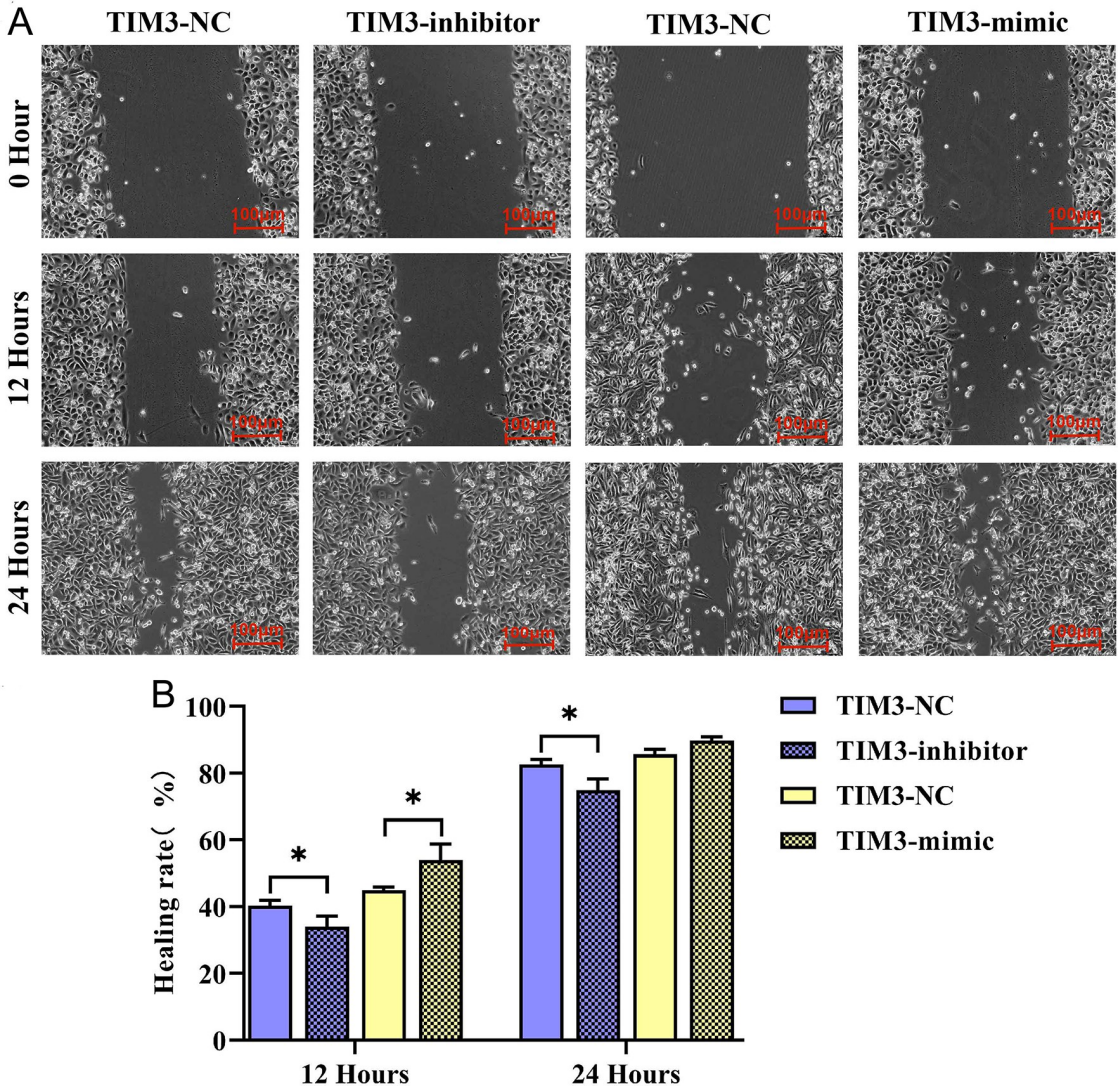
TC cell migrations was detected by scratch assay. A: Cell scratch assay showed the migration ability of TC cells in TIM3-inhibitor and TIM3-mimic groups at 12 h and 24 h (×400); B: The results of cell scratch migration of TIM3-inhibitor and TIM3-mimic groups at 12 h and 24 h were statistically analyzed. * represents *p* < 0.05.

**Fig 6:**
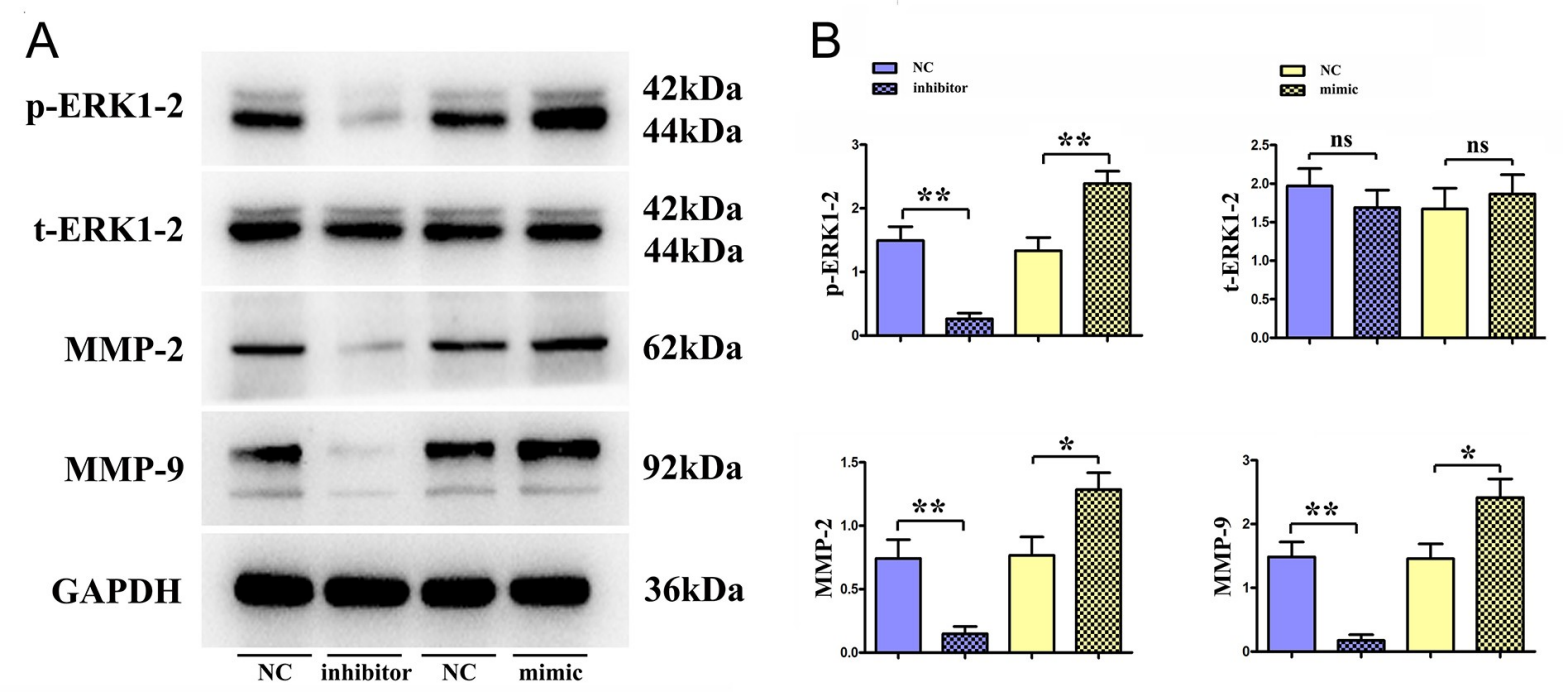
Western blotting was performed to detect the expression of p-ERK1/2, t-ERK1/2, MMP-2 and MMP-9 in TC cell of TIM3-inhibitor and TIM3-mimic groups. A: Protein band pattern of p-ERK1/2, t-ERK1/2, MMP-2, and MMP-9; B: The statistical analysis of protein expression of p-ERK1/2, t-ERK1/2, MMP-2, and MMP-9. * represents *p* < 0.05; ** represents *p* < 0.01. Ns: not significant.

The effects of TIM3 on the migratory and invasive abilities of TC cells were detected by Transwell assay (Fig 4) and wound scratch assay (Fig 5), respectively. Results of wound scratch assay showed that the width of cell scratch in TIM3-mimic group was significantly smaller than that in TIM3-NC group at 12 and 24 h, suggesting that the distance of tumor cells migrating toward the scratch center is increased remarkably. On the contrary, the width of cell scratch was overtly increased in TIM3-inhibitor group compared with that in TIM3-NC group. Similarly, it was indicated in the results of Transwell assay that in contrast with those in TIM3-NC group, the numbers of migrating TC cells and basement membrane-penetrating cells were raised evidently in TIM3-mimic group, while they were reduced obviously in TIM3-inhibitor group. Consistently, the results of Western blotting assay (Fig 6) revealed that TIM3-mimic group had notably higher protein expressions of tumor invasion and metastasis-related molecules, matrix metalloproteinase-2 (MMP-2) and MMP-9 in TC cells than TIM3-NC group. These results indicate that TIM3 is able to enhance the migratory and invasive abilities of TC cells.

### TIM3 affected migratory, invasive abilities of TC cells through activating the ERK1/2 pathway

**Fig 7:**
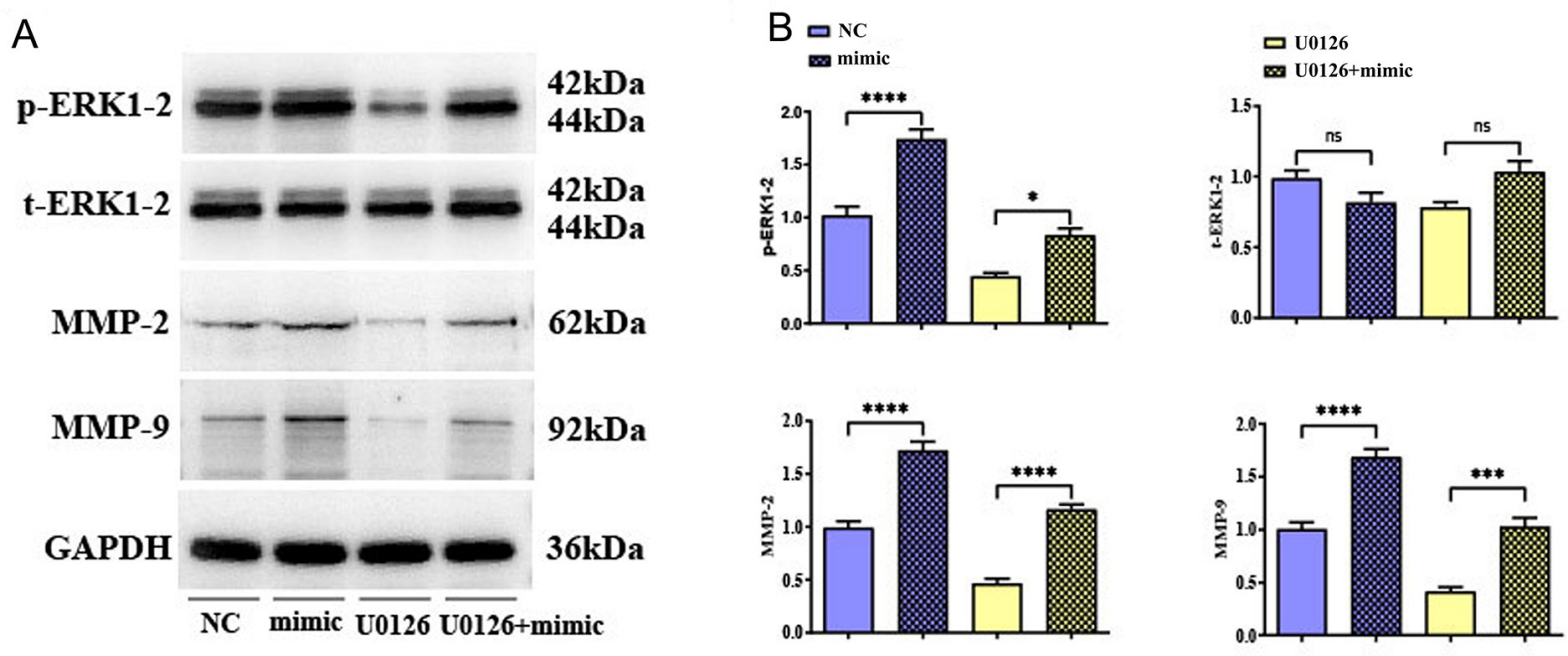
Western blotting was performed to detect the expression of p-ERK1/2, t-ERK1/2, MMP-2 and MMP-9 in TC cell of U0126 and mimic+U0126 groups. A: Protein band pattern of p-ERK1/2, t-ERK1/2, MMP-2, and MMP-9; B: The statistical analysis of protein expression of p-ERK1/2, t-ERK1/2, MMP-2, and MMP-9. * represents *p* < 0.5, *** represents *p* < 0.001, **** represents *p* < 0.0001. Ns: not significant.

Based on the aforementioned experimental results, TIM3 is capable of advancing the migratory, invasive and proliferative abilities of TC cells. In addition, based on BP analysis of GO terms, 316 common DEGs were involved in ERK1 and ERK2 cascade, regulation of ERK1 and ERK2 cascade, positive regulation of ERK1 and ERK2 cascade, and negative regulation of ERK1 and ERK2 cascade. Therefore, the effect of TIM3 on p-ERK1/2 protein expression was analyzed. The results of Western blotting assay (Fig 6) revealed that TIM3-mimic group had notably higher protein expressions of p-ERK1/2 in TC cells than TIM3-NC group. In combination with the above results, the protein expression of ERK1/2 was suppressed using the inhibitor U0126 to investigate the specific molecular mechanism of TIM3 in affecting the migratory, invasive and proliferative abilities of TC cells. The results of Western blotting assay (Fig 7) revealed that mimic+U0126 group had notably higher protein expressions of p-ERK1/2, MMP-2, MMP-9 in TC cells than U0126 group. These results indicate that TIM3 may affect the migratory, invasive and proliferative abilities of TC cells by activating the ERK1/2 pathway.

## Discussion

TC has shown a rising incidence and death rates in recent years [16], which are doubled in females compared with those in males [17]. Among various types of TC, papillary thyroid carcinoma (PTC) occurs the most frequently [18]. Constant exploration and research of integrative genomics revealed that many genes and proteins have correlations with the carcinogenesis of TC, including the mutations (BRAF, RAS and RET/PTC) related to invasiveness (metastasis and recurrence) [19].

In the present study, it was found that TIM3 could strengthen the migratory, invasive and proliferative abilities of TC cells. TIM3 plays an important regulatory role in tumor immunity [20]. The down-regulation of TIM3 represses the proliferation, migration and invasion as well as induces the apoptosis of tumor cells [10]. It was manifested by the results of wound scratch assay in this study that the width of cell scratch in TIM3-mimic group was significantly smaller than that in TIM3-NC group at 12 and 24 h, suggesting that the distance of tumor cells migrating toward the scratch center is increased remarkably. On the contrary, the width of cell scratch was overtly increased in TIM3-inhibitor group. Similarly, it was indicated in the results of Transwell assay that in contrast with those in TIM3-NC group, the numbers of migrating TC cells and basement membrane-penetrating cells were raised evidently in TIM3-mimic group, while they were reduced obviously in TIM3-inhibitor group. Consistently, the results of Western blotting assay revealed that TIM3-mimic group had notably higher protein expressions of MMP-2 and MMP-9 in TC cells than TIM3-NC group. MMP-2 and MMP-9 all belong to the MMP family. MMPs are a category of zinc enzymes capable of degrading all extracellular matrix components, which is a crucial event in the invasion and metastasis of the majority of tumor cells [21]. Under normal conditions, MMPs are involved in wound repair [22]. Furthermore, MMPs may participate in multiple carcinogenesis processes, including the migration, invasion and metastasis of cancer cells [23]. There are cysteine insertions in the catalytic domain of MMPs, which are usually shielded by the propeptide region of the structure in a physiological resting state. When MMPs are activated, these insertions can conjugate with and lyse gelatin, collagen and elastin, degrade the extracellular matrix, regulate adhesion between tumor cells and promote angiogenesis and cell invasion [24]. In TC, the increased expression of MMP2 facilitates cell invasion and associated with lymph node metastasis [25, 26]. Epigenetic regulation of the MMP9 is related to the tumor migration suppression in PTC [27]. Hence, TIM3 can improve the migratory and invasive abilities of TC cells.

The action mechanism of TIM3 in TC cells is worthy of investigation. The protein expression of ERK1/2 was suppressed using the inhibitor U0126 to investigate the specific molecular mechanism of TIM3 in affecting the migratory, invasive and proliferative abilities of TC cells. The results of Western blotting assay revealed that mimic+U0126 group had notably higher protein expressions of p-ERK1/2, MMP-2, MMP-9 in TC cells than U0126 group. ERK1 and ERK2, the members of the mitogen-activated protein kinase (MAPK) super family, modulate cell cycle and apoptosis. It is reported that the combination of ERK1/2 inhibitors and BRAF significantly inhibits the growth of TC cells and reduces the activity of the MARK signaling pathway [28]. In this study, our results indicate that TIM3 may activate the ERK1/2 pathway to affect the migratory, invasive abilities of TC cells.

Based on PPI analysis, we found that 10 genes had an interaction with TIM3, including PRDM1, ITGAX, CD27, IL18, CD68, CD4, ENTPD1, FCGR3A, LCP2, and FYN. ITGAX is expressed in various TC cell lines [29]. PRDM1 is associated with overall survival of TC patients [29]. The percentage of CD4+ T cells is lower in patients with advanced metastatic TC [30]. ENTPD1 is highly expressed in TC, and high expression of ENTPD1contributs to the prognosis of TC patients [31]. IL18 and FYN are involved in PTC [32, 33]. CD27 is associated with the metastasis risk in medullary thyroid cancer (MTC) [34]. A significant over expression of FCGR3A and LCP2 is found in anaplastic thyroid carcinoma (ATC) [35, 36]. It is suggested that the interaction between TIM3 and these genes may be associated with the invasion and migration of TC.

In this study, we have demonstrated that TIM3 may affect the migratory and invasive of TC cells by activating the ERK1/2 pathway, which may provide a novel field in understanding the molecular mechanism of TIM3 in TC. Moreover, the migration and invasion of TC cells can be inhibited by reducing TIM3 expression, which may be helpful for clinical patient management. In addition, the effect of TIM3 on the ERK1/2 pathway could also be used to guide clinical trials of drug therapy for advanced TC.

However, there are limitations to our study. Firstly, interaction between TIM3 and 10 genes is needed to validate in the immunoprecipitation assay. Secondly, some other enriched signaling pathways are needed to be explored in further study.

## Supporting information

S1 Checklist Human participants research checklist. (DOCX)

S1 Table Primer sequences were used in this study. (DOCX)

S1 Fig Detailed details of overexpression vector. (TIF)

## List of abbreviations

ATC: anaplastic thyroid carcinoma
CC: cell component
CD27: CD27 molecule
CD4: CD4 molecule
CD68: CD68 molecule
DAVID: Database for Annotation, Visualization and Integration Discovery
DEGs: Differentially expressed genes
DMEM: Dulbecco’s modified Eagle medium
ENTPD1: ectonucleoside triphosphate diphosphohydrolase 1
FBS: fetal bovine serum
FC: fold change
FCGR3A: Fc gamma receptor IIIa
FYN: FYN proto-oncogene
GEO: Gene Expression Omnibus
GO: Genome Ontology
GSEA: gene set enrichment analysis
IL18: interleukin 18
ITGAX: integrin subunit alpha X
KEGG: Kyoto Encyclopedia of Genes and Genomes
LCP2: lymphocyte cytosolic protein 2
MAPK: mitogen-activated protein kinase
MF: molecular function
MMP-2: matrix metalloproteinase-2
MTC: medullary thyroid cancer
NC: negative control
PBS: phosphate-buffered saline
PCA: principal component analysis
PPI: Protein-protein interaction
PRDM1: PR/SET domain 1
PTC: papillary thyroid carcinoma
TC: thyroid carcinoma
TIM3 T: cell immunoglobulin and mucin domain 3
