# Supplementary material for: TIM3 activates the ERK1/2 pathway to promote invasion and migration of thyroid tumors
Source: PLoS One. 2024 Apr 3;19(4):e0297695. doi: 10.1371/journal.pone.0297695 (PMC10990238; doi:10.1371/journal.pone.0297695)
Supplement: S1 Table — (DOCX) [file pone.0297695.s002.docx]

**S1 Table: Primer sequences were used in this study**

| **Primer name** | **Primer sequence** |
| --- | --- |
| pcDNA3.1-TIM3-F | 5’-TACCGAGCTCGGATCCATGTTTTCACATCTTCCCTTTG-3’ |
| pcDNA3.1-TIM3-R | 5’-TGGATATCTGCAGAATTCCTATGGCATTGCAAAGCGAC-3’ |
| TIM3-siRNA-F | 5’-CAUGGAUGUUAGAGCUCAA-3’ |
| TIM3-siRNA-R | 5’-UUGAGCUCUAACAUCCAUG-3’ |
| TIM3-NC-F | 5’-CAUGGAUGUUUGAGCUCAA-3’ |
| TIM3-NC-R | 5’-CAUGGAUGUUAGUGCUCAA-3’ |
