# Supplementary figures and images for: TIM3 activates the ERK1/2 pathway to promote invasion and migration of thyroid tumors
Source: PLoS One. 2024 Apr 3;19(4):e0297695. doi: 10.1371/journal.pone.0297695 (PMC10990238; doi:10.1371/journal.pone.0297695)

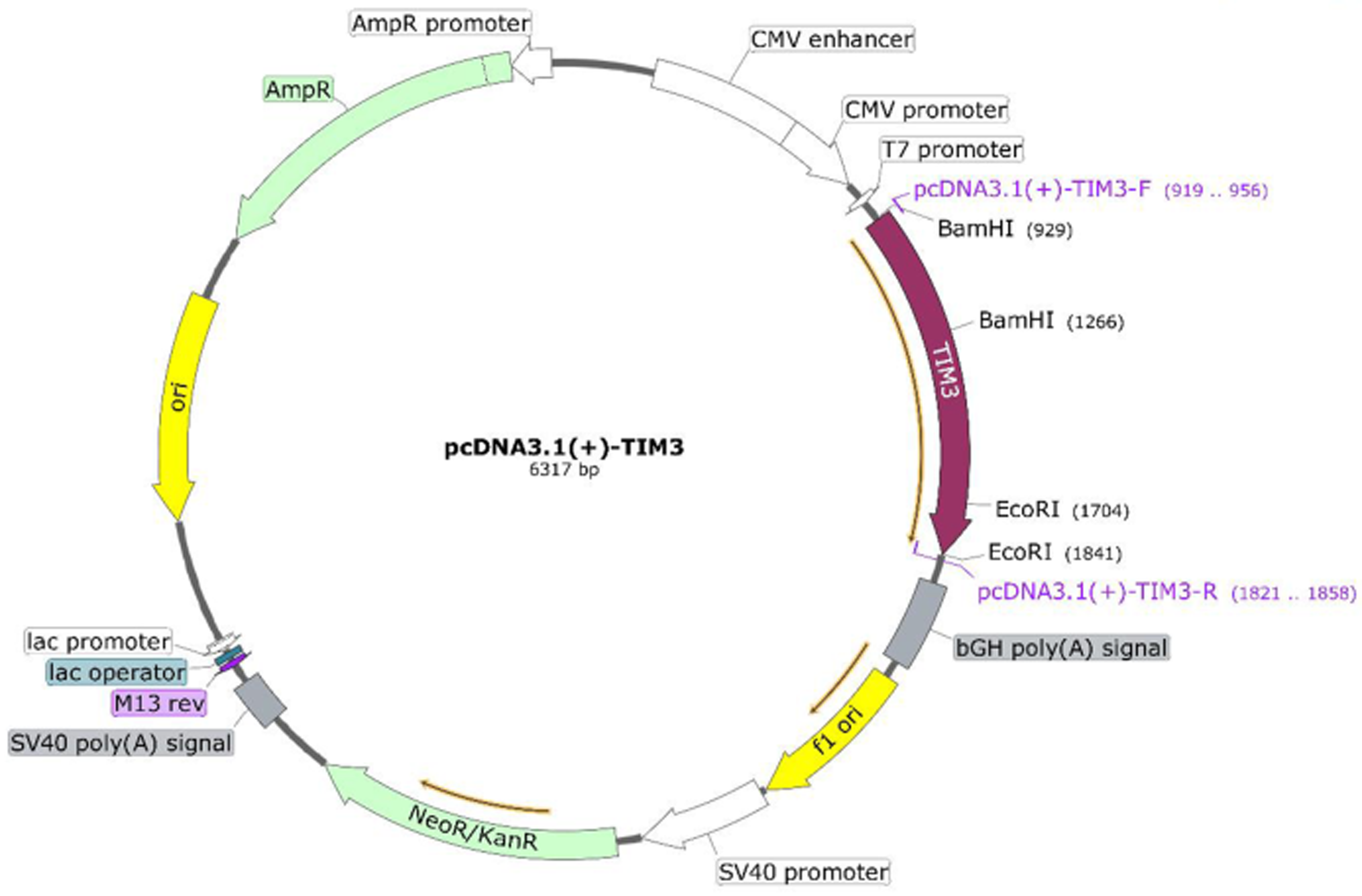

Supplement: S1 Fig — (TIF) [file pone.0297695.s003.tif]
